# Genetic evolution of Marek’s disease virus in vaccinated poultry farms

**DOI:** 10.14202/vetworld.2021.1342-1353

**Published:** 2021-05-28

**Authors:** Nahed Yehia, Hemat S. El-Sayed, Sabry E. Omar, Ahmed Erfan, Fatma Amer

**Affiliations:** 1 Reference Laboratory for Veterinary Quality Control on Poultry Production, Animal Health Research Institute, Agricultural Research Center, Dokki, Giza 12618, Egypt; 2 Department of Poultry Diseases, Benha Provincial Laboratory, Animal Health Research Institute, Agricultural Research Center, Giza, Egypt

**Keywords:** genetic characterization, *gL*, *ICP4*, marek’s disease virus, *Meq*

## Abstract

**Background and Aim:**

The Marek’s disease virus (MDV) is a neoplastic disease causing serious economic losses in poultry production. This study aimed to investigate MDV occurrence in poultry flocks in the Lower Egypt during the 2020 breakout and genetically characterized *Meq*, *gL*, *and ICP4* genes in field strains of MDV.

**Materials and Methods:**

Forty samples were collected from different breeds from eight Egyptian governorates in 2020. All flocks had received a bivalent vaccine (herpesvirus of turkey FC-126 + Rispens CVI988). However, weight loss, emaciation, reduced egg production, paralysis, and rough/raised feather follicles occurred. Samples were collected from feather follicles, liver, spleen, and nerve tissue for diagnosis by polymerase chain reaction. MDV genetic characterization was then performed by sequencing the *Meq*, *gL, and ICP4* genes of five positive samples representing different governorates and breeds.

**Results:**

A total of 28 samples were positive for MDV field strains, while two were related to MDV vaccinal strains. All samples tested negative for ALV (A, B, C, D, and J) and REV. Phylogenetic analysis of the *Meq* gene of sequenced samples revealed that all MDVs were related to the highly virulent European viruses (Gallid herpesvirus 2 ATE and PC12/30) with high amino acid (A.A.) identity 99.2-100%. Alternatively, there was low A.A. identity with the vaccine strains CVI988 and 3004 (up to 82.5%). These results indicate that further investigation of the efficacy of current Egyptian vaccines is required. The Egyptian strains also harbor a specific mutation, allowing clustering into two subgroups (A and B). By mutation analysis of the *Meq* gene, the Egyptian viruses in our study had R101K, P217A, and E263D mutations present in all Egyptian viruses. Furthermore, R176A and T180A mutations specific to our strains contributed to the high virulence of highly virulent strains. There were no mutations of the *gL* or *ICP4* genes.

**Conclusion:**

Further studies should evaluate the protection contributed by current vaccines used in Egypt.

## Introduction

Marek’s disease virus (MDV) is a highly oncogenic, lymphoproliferative, and neuropathic disease. It is an economically devastating infectious disease for the poultry industry due to emaciation, lower egg production, and carcass condemnations [1,2]. It is also commonly identified in domestic chickens and more rarely in turkey and quail species [3]. The disease manifests in chickens 3-4 weeks of age or older and is usually diagnosed at 12 and 30 weeks of age [4]. The infection is airborne and horizontally transmitted to other chickens. Although vertical transmission to offspring occurs, it is rare [5]. MDV was widespread globally, and as a disease threat, continues to be an important area of research [6]. A major concern is the continued evolution of the virus and the emergence of more virulent MDV pathotypes despite an extensive vaccination regime in the poultry industry [7]. There are three MDV serotypes (MDV-1, 2, and 3), which have major differences between their genomes and biological features [8,9]. MDV-1 includes all virulent oncogenic strains and their attenuated forms used as a vaccine. MDV-2 includes the naturally avirulent, nonpathogenic, and non-oncogenic strain isolated in chickens, some of which are used as vaccines. MDV-3 includes the herpesvirus of turkeys (HVTs) [10]. Only MDV-1 strains cause disease in chickens. The MDV-1 strains are the most commonly used for vaccination, whereas avirulent MDV-2 and MDV-3 strains have also been exploited for vaccine development. MDV-2 strains have been employed, particularly for bivalent vaccines, together with HVT [4]. Alternatively, there are four MDV-1 pathotypes, including mild (m), virulent (v), very virulent (vv), and very virulent plus (vv+) strains [11]. Many MDV sequences have also been described in various countries [12-14].

MDV replicates in B and T lymphocytes cause lymphoma of the peripheral nervous system, visceral organs, and skin [15,16]. Despite the intensive CVI988 vaccination policy [10], MDV infection will still spread in poultry. This prediction is due to acquired mutations contributing to increased virulence [17-19], mishandling or incorrect storage of the vaccine, or the presence of immunosuppressive diseases [20].

The MDV genome is a double-stranded DNA structure of about 175 kb, consisting of long (U.L.) and short (U.S.) unique regions, each flanked by inverted repeats (TRL, IRL, IRS, and TRS) [21]. All MDV serotypes have the same general structure, but serotype I contains unique genes found in the repeated regions of the genome, including the *Meq* oncogene and *pp38, VIL8, and vTR* [22]. The *Meq* gene is expressed abundantly in MDV-infected cells and MDV tumor cells and plays an important role in the transformation process [23]. The *Meq* gene has also been studied as a potential causal factor for the high oncogenicity of the virus [13,14,24]. Losses or deletion of the *Meq* gene leads to loss of oncogenicity as recorded in MD-2 and MD-3 strains [21,25], although other genes also serve important roles in lymphoma development [20].

MDV contains ten glycoproteins (*gL*, *gM*, *gH*, *gB*, *gC*, *gN*, *gK*, *gD*, *gI*, and *gE*), and is encoded by *UL1*, *UL10*, *UL22*, *UL27*, *UL44*, *49.5 UL53*, *US6*, and *US7*, respectively [13]. While the gH and *gL* proteins form a hetero-oligomeric complex [26], which plays a significant role in the viral entry of the host cell, *gL* and *gC* exhibit an important role in the development of a cytotoxic immune response [12]. Recent evidence associates a four–amino acid (A.A.) deletion within the putative signal cleavage site of *gL* with increased virus virulence [13]. Numerous studies conducted in Egypt have also investigated the epidemiology of the MDV strains circulating in Egypt about outbreaks in poultry flocks [27,28]. However, minimal data regarding the molecular evolution of MDV strains circulating in Egypt are available.

This study aimed to investigate the current situation of tumor viruses in eight governorates in Lower Egypt during 2020. The study also investigates the genetic evolution of *Meq*, *gL*, and *ICP4* genes in field viruses of MDV recently detected from infected vaccinated flocks.

## Materials and Methods

### Ethical approval

This study does not require the approval of the Institute Animal Ethics Committee

### Study period and location

We collected forty samples from January 2020 to December 2020. The samples were processed in Reference Laboratory for Veterinary Quality Control on Poultry Production, Animal Health Research Institute. **-**

### Sampling

Forty samples from eight governorates were collected from the Lower Egypt and from different breeds (layer and breeder farms). Twenty to thirty birds from each flock were checked. Samples of the 3-4 feather follicles and liver, spleen, and nerves showing gross pathologic lesions indicative of MDV were taken from individual birds. The samples from each farm were pooled and tested as a single sample. All flocks had received the bivalent vaccine (HVT FC-126 + Rispens CVI988). The tissue was also homogenized using Qiagen tissue lyser, suspended in sterile PBS, and centrifuged at 3000 rpm at 4°C for 15 min to get the supernatants. The specimens were stored at −20°C until use. DNA extraction was performed, after which gross pathological lesions were confirmed on necropsy.

### DNA extraction for PCR

The total nucleic acid extraction was performed using the QIAamp mini elutes virus spin kit (Qiagen, Germany, GmbH). In brief, 200 μL of the tissue homogenate supernatant was incubated at 56°C for 15 min using a 25 μL Qiagen protease and 200 mL A.L. lysis buffer. The remaining steps were completed according to the manufacturer’s instructions.

### Amplification of MDV, ALV (A, B, C, D, and J), and REV DNA

Tumor viruses were detected using the Phusion^®^ high fidelity DNA polymerase (Thermo, MA, USA) and specific primers for MDV and other tumor viruses {ALV (A, B, C, D, and J) and REV} [29-34]. Furthermore, the differentiation between field and vaccinal strains of MDV was achieved using (BamH1 132 bp tandem repeat) primers [29] (Supplementary Table-.1) according to the manufacturer’s instructions. Specific DNA amplicons were then identified by agarose gel electrophoresis, and a gel documentation system (Alpha Innotech, Biometra, Germany) was used to capture gel photosystem.

**Supplementary Table-1:** Parameter of PCR amplification of tumor viruses.

Target gene	Primers sequences	Amplified segment (bp)	Primary denaturation	Amplification (35 cycles)			Final extension	Reference

				Secondary denaturation	Annealing	Extension		
*MDV-meq*	M-S ATGTCTCAGGAGCCAGAGCCGGCGCT MR-AS GGGGCATAGACGATGTGCTGCTGAG	1062	94°C 5 min	94°C 30 s	57°C 40 s	72°C 45 s	72°C 10 min	[29]
MDV-BamH1-H 132 bp tandem repeat	M1-F TACTTCCTATATAGATTGAGACGT M2-R GAGATCCTCGTAAGGTGTAATATA	434	94°C 5 min	94°C 30 s	55°C 40 s	72°C 45 s	72°C 10 min	[30]
MDV-ICP4	MDV-1.1 GGA TCG CCC ACC ACG ATT ACT ACC MDV-1.8 ACT GCC TCA CAC AAC CTC ATC TCC	247	94°C 5 min	94°C 30 s	55°C 30 s	72°C 30 s	72°C 7 min	[31]
MDV-GL	MDV-GL-F ATG AAA ATT TAT AGA GTA CTC GTG MDV-GL-R GGC ATT GGC TCG TCG GCT	586	94°C 5 min	94°C 30 s	50°C 30 s	72°C 30 s	72°C 7 min	[32]
ALV A	H5-F GGATGAGGTGACTAAGAAAG EnvA-R AGAGAAAGAGGGGYGTCTAAGGAGA	694	94°C 5 min	94°C 30 s	48°C 30 s	72°C 30 s	72°C 7 min	[33]
ALV-B and D	BD-F CGAGAGTGGCTCGCGAGATGG BD-R AGCCGGACTATCGTATGGGGTAA	1100	94°C 5 min	94°C 30 s	52°C 30 s	72°C 30 s	72°C 7 min	[34]
ALV-C	C-F CGAGAGTGGCTCGCGAGATGG C-R CCCATATACCTCCTTTTCCTCTG	1400	94°C 5 min	94°C 30 s	52°C 30 s	72°C 30 s	72°C 7 min	[35]
ALV-J	H5-F GGATGAGGTGACTAAGAAAG H7-R CGAACCAAAGGTAACACACG	545	94°C 5 min	94°C 30 s	48°C 30 s	72°C 30 s	72°C 7 min	[36]
REV	env-F AGCTAGGCTCGTATGAA env-R TATTGACCAGGTGGGTTG	438	94°C 5 min	94°C 30 s	48°C 30 s	72°C 30 s	72°C 7 min	[37]

The table shows Primers sequences, target genes, amplicon sizes, and cycling conditions. MDV=Marek disease virus

### DNA sequencing of *Meq, gL, and ICP4* genes of MDV

Representative positive samples of different origins were selected for the molecular characterization of *Meq*, *gL, and ICP4* genes. The Phusion® high fidelity DNA polymerase (Thermo, MA, USA) and primers specific to each gene [35-37] were used according to the manufacturer’s instructions (Supplementary Table-1).

Purification of PCR product was then conducted using the QIAquick Gel Extraction Kit (Qiagen, Hilden, Germany), while sequencing was conducted using the BigDye Terminator v3.1 Cycle Sequencing Kit (Applied Biosystems, California, USA) with gene-specific primers. Nucleotide sequences were also obtained using an ABI 3500 Genetic Analyzer (Life Technologies, California, USA). The sequences in this study were published with specific accession numbers in The National Center for Biotechnology Information.

### Genetic and phylogenetic analysis

DNA and A.A. sequences were aligned with 20 related strains from Europe, China, the USA, and Egypt GenBank using the MegAlign module of the DNASTAR software (Lasergene; version 7.2; DNASTAR, Madison, WI, USA). The phylogenetic tree was built using MEGA (version 7; www.megasoftware.net) with moderate strength, and 1000 bootstrap replicates using a maximum likelihood tree method. The pair-wise nucleotide percent identity was also performed using DNASTAR software.

## Results

### Clinical signs

The observed clinical signs in all affected flocks were loss of weight, emaciation, and reduced egg production in layers. Twenty of the affected flocks had neural lesions (development of paralysis of the legs, wings, and neck). The other ten affected flocks had neural lesions and visceral tumors, while ten other flocks showed visceral tumors only, with 60% of affected birds displaying rough and raised feather follicles.

### Gross pathology

All affected birds showed loss of striations and thickening of their nerve trunk. Twenty of the affected flocks showed gray-white foci neoplastic tissues in the visceral organs, including the liver, kidney, spleen, gonads, heart, lungs, and skeletal muscles.

### Polymerase chain reaction

Based on the 434-bp positive amplification of the Bam1H-H132 bp tandem repeats, 30 samples were identified as positive for MDV from different breeds (28 field strain and two vaccinal strains). All other tested samples were negative for ALV (A, B, C, and D) and REV (Table-1).

**Table-1:** Epidemiological data of collected samples, clinical signs, and result of PCR.

No.	Year	Breed	Production	Governorate	Clinical Signs	Result of PCR BamH1-H
1.	1/2020	High line	Layer	Dakahleya	Loss of weight, emaciation, reduced egg production, paralysis of the legs	Positive MDV (field strain)
2.	5/2020	Avian	Breeders	Sharkia	Loss of weight, emaciation, reduced egg production, paralysis of the legs, wings and neck	Positive MDV (field strain)
3.	3/2020	High line	Layer	Gharbeya	Loss of weight, emaciation, reduced egg production, paralysis of the legs, wings and neck	Negative
4.	2/2020	High line	Layer	Kaliobeya	Loss of weight, emaciation, reduced egg production, paralysis of the legs, wings and neck, paralysis of the legs, wings and neck	Positive MDV (field strain)
5.	5/2020	H&N	Layer	Kafr elsheikh	Loss of weight, emaciation, reduced egg production, paralysis of the legs, wings and neck	Positive MDV (field strain)
6.	8/2020	Cobb	Breeder	Gharbeya	Loss of weight, emaciation, reduced egg production, paralysis of the legs, wings and neck	Positive MDV (field strain)
7.	9/2020	H&N	Layer	Giza	Loss of weight, emaciation, reduced egg production, paralysis of the legs, wings, and neck	Positive MDV (field strain)
8.	2/2020	High line	Layer	Giza	Loss of weight, emaciation, reduced egg production, paralysis of the legs, wings, and neck	Positive MDV (field strain)
9.	7/2020	Cobb	Breeder	Behira	Loss of weight, emaciation, reduced egg production paralysis of the legs, wings and neck	Positive MDV (field strain)
10.	11/2020	Red ISA	Breeder	Sharkia	Loss of weight, emaciation, reduced egg production paralysis of the legs, wings, and neck	Negative
11.	12/2020	Dokki 4	Layer	Sharkia	Loss of weight, emaciation, reduced egg production, paralysis of the legs, wings, and neck	Positive MDV(field strain)
12.	7/2020	Novogen	Layer	Behira	Loss of weight, emaciation, reduced egg production, paralysis of the legs, wings, and neck	Positive MDV (field strain)
13.	8/2020	Avian	Layer	Kaliobeya	Loss of weight, emaciation, reduced egg production, rough and raised feather follicles, paralysis of the legs, wings and neck	Negative
14.	10/2020	Hubbard	Broiler	Behira	Loss of weight, emaciation , reduced egg production, rough and raised feather follicles	Positive MDV (field strain)
15.	1/2020	Baladi	Layer	Sharkia	Loss of weight, emaciation, reduced egg production, rough and raised feather follicles, paralysis of the legs, wings, and neck	Positive MDV (vaccinal strain)
16.	5/2020	High line	Layer	Dakahleya	Loss of weight, emaciation, reduced egg production, rough and raised feather follicles, paralysis of the legs, wings, and neck	Negative
17.	9/2020	Hubbard	Broiler	Kaliobeya	Loss of weight, emaciation, reduced egg production, rough and raised feather follicles, paralysis of the legs, wings, and neck	Negative
18.	11/2020	Red ISA	Breeder	Behira	Loss of weight, emaciation, reduced egg production, paralysis of the legs, wings, and neck	Positive MDV (field strain)
19.	8/2020	High line	Layer	Dakahleya	Loss of weight, emaciation, reduced egg production, paralysis of the legs, wings, and neck	Positive MDV (field strain)
20.	4/2020	H&N	Layer	Giza	Loss of weight, emaciation, reduced egg production, paralysis of the legs, wings, and neck	Positive MDV (field strain)
21.	6/2020	Avian	Broiler	Giza	Loss of weight, emaciation, reduced egg production, paralysis of the legs, wings, and neck	Positive MDV (field strain)
22.	4/2020	High line	Layer	Behira	Loss of weight, emaciation, reduced egg production paralysis of the legs, wings, and neck	Positive MDV (field strain)
23.	9/2020	Cobb	Broiler	Dakahleya	Loss of weight, emaciation, reduced egg production paralysis of the legs, wings, and neck	Positive MDV (field strain)
24.	12/2020	Baladi	Layer	Kaliobeya	Loss of weight, emaciation, reduced egg production, rough and raised feather follicles, paralysis of the legs, wings, and neck	Positive MDV (field strain)
25.	3/2020	High line	Layer	Behira	Loss of weight, emaciation, reduced egg production, rough and raised feather follicles	Positive MDV (field strain)
26.	7/2020	H&N	Layer	Kaliobeya	Loss of weight, emaciation, reduced egg production, rough and raised feather follicles	Positive MDV (field strain)
27.	2/2020	Cobb	Breeder	Sharquia	Loss of weight, emaciation, reduced egg production	Negative
28.	6/2020	Cobb	Breeder	Kaliobeya	Loss of weight, emaciation , reduced egg production	Positive MDV (field strain)
29.	12/2020	Avian	Breeder	Giza	Loss of weight, emaciation, reduced egg production, rough and raised feather follicles, paralysis of the legs, wings and neck	Positive MDV (field strain)
30.	11/2020	High line	Layer	Behira	Loss of weight, emaciation, reduced egg production, raised feather follicles, paralysis of the legs, wings and neck	Negative
31.	4/2020	Cobb	Broiler	Sharquia	Loss of weight, emaciation and reduced egg production, rough, raised feather follicles, paralysis of the legs, wings, and neck	Positive MDV (field strain)
32.	12/2020	H&N	Layer	Behira	Loss of weight, emaciation, reduced egg production	Negative
33.	7/2020	Avian	Breeder	Giza	Loss of weight, emaciation and reduced egg production	Positive MDV (field strain)
34.	9/2020	High line	Layer	Kafr elsheikh	Loss of weight, emaciation, reduced egg production, rough and raised feather follicles	Positive MDV (field strain)
35.	6/2020	Baladi	Layer	Gharbeya	Loss of weight, emaciation, reduced egg production, rough and raised feather follicles	Negative
36.	3/2020	Cobb	Breeder	Behira	Loss of weight, emaciation, reduced egg production	Positive MDV (field strain)
37.	8/2020	Cobb	Breeder	Giza	Loss of weight, emaciation, reduced egg production, rough and raised feather, paralysis of the legs, wings and neck follicles	Positive MDV (field strain)
38.	10/2020	High line	Layer	Kafr elsheikh	Loss of weight, emaciation, reduced egg production, rough and raised feather follicles, paralysis of the legs, wings, and neck	Positive MDV (field strain)
39.	8/2020	H&N	Layer	Sharquia	Loss of weight, emaciation, reduced egg production, rough, raised feather follicles, paralysis of the legs, wings and neck	Positive MDV (vaccinal strain)
40.	9/2020	Cobb	Breeder	Behira	Emaciation, reduced egg production, rough, raised feather follicles, paralysis of the legs, wings and neck	Negative

The table shows governorates, breeds, type of production, and year of collected samples and clinical signs observed and result of PCR. MDV=Marek disease virus

### Molecular characterization of *Meq* gene in Egyptian viruses

**Figure-1:**
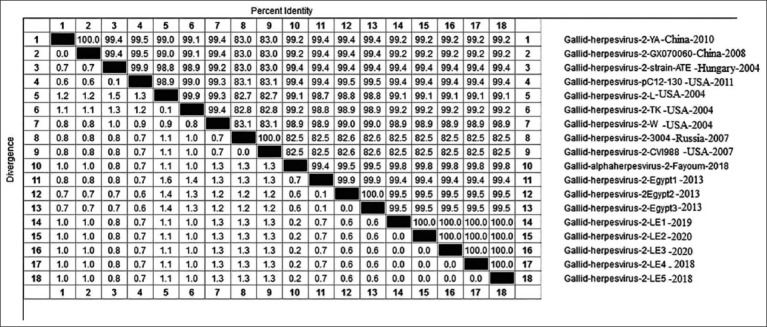
Amino acid (A.A.) identities and divergence of *Meq* gene of sequenced viruses compared to other selected strains from European, china and American strains. The figure shows comparative alignment of *Meq* gene showed that *Meq* A.A. identity percent of 99.2-99.4% with European and china strains YA, ATE, PC12/130, and 99.4-99.8% and with other Egyptian and 82.5% with vaccinal strains CV1988 and 3004.

Phylogenetic analysis indicated that the five samples from field samples identified in this study were genetically related to the virulent European and Chinese viruses (Y.A., ATE, PC12/130, and GX070060) with a 99.2-99.4% homology. The viruses in this study also shared a 99.4-99.8% homology and with other Egyptian viruses. They also shared a homology of 82.5% with the vaccinal strains; CV1988 and 3004 (Figure-1). All viruses in this study had short *Meq* sequences than the mild and vaccinal strains, with 177-bp length. A.A. mutations were also detected in the *Meq*_Egypt strains compared with the Gallid herpesvirus 2 ATE strain at R101K, and P217A as Egyptian viruses, Chinese viruses (LMS, Y.A., WS03, and GX070060), and USA (TK, X, and N), respectively. The E263D mutation was specific for all Egyptian strains. R176A and T180A were also specific to Egyptian viruses in this study.

**Figure-2:**
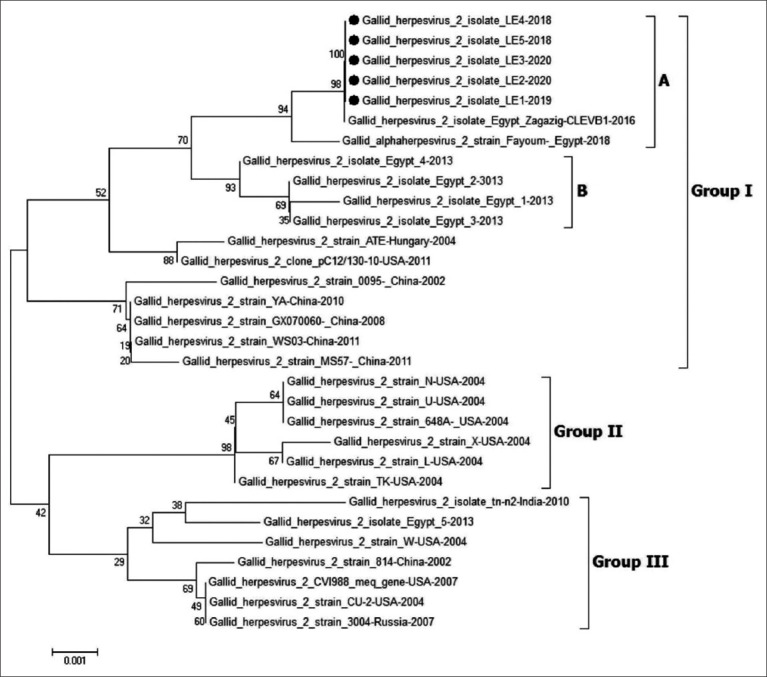
Phylogenetic tree of *Meq* gene of Marek disease virus (MDV). The figure shows The phylogenetic analysis of *Meq* gene of MDV gene reveling that all Egyptian strains cluster were related to very virulent European strains (ATE, PC12/130) and cluster into two subgroups (A and B). The MDV viruses in our study are indicated with a black dot.

The A.A. sequences of the *Meq* gene were downloaded from GenBank for 20 reference strains. Then, they were aligned with the strains presently studied, revealing that the *Meq* gene phylogenetic tree is classified into three groups. Group I contained the Chinese viruses (LMS, Y.A., WS03, and GX070060) and the European viruses (AT-2539 and PC12/130). The Egyptian viruses were also closely related to the vv European viruses and divided into two subgroups (a and b; Figure-2). The MDV viruses of this study were also related to subgroup A due to a specific characteristic nucleotide mutation. Alternatively, Group II was related to the virulent strains and vv viruses isolated from the USA (TK, X, and N). Group III comprised mildly virulent viruses and vaccinal strains (CV1988, CU-2, 814, and 3004) (Figure-2).

### Molecular characterization of the *gL* gene in Egyptian viruses

**Figure-3:**
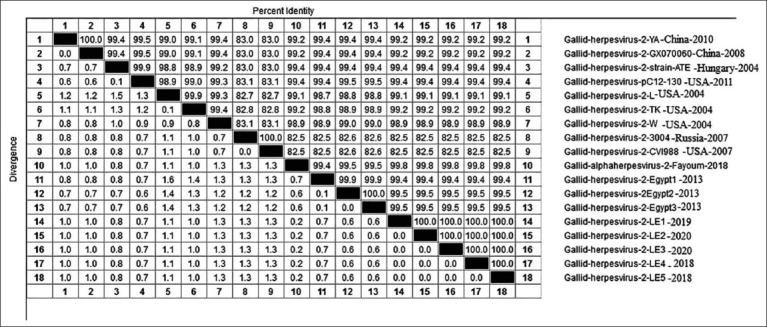
Amino acid (A.A.) identities and divergence of *gL* gene of sequenced viruses compared to other selected strains from European, China, and American strains. The figure shows comparative alignment of *Meq* gene showed that *gL* A.A. identity percent of 99.7% with European and china strains YA, ATE, PC12/130 and 99.7% A.A. identity with other Egyptian strains, mildly virulent strain, and vaccinal strain.

Phylogenetic analysis indicated that *gL* in the MDV strain studied was genetically similar to that of the vv European and Chinese viruses (LMS, ATE, and PC12/130) with a 99.7% homology. The A.A identity percentage also reached 96.9-99.7% with the TK and N strains, and 99.7% with the other Egyptian viruses, mildly virulent viruses, and the vaccinal strains; CV1988 and CU-2 (Figure-3). No mutation was detected in comparison to the Gallid herpesvirus-2 strain ATE from China.

**Figure-4:**
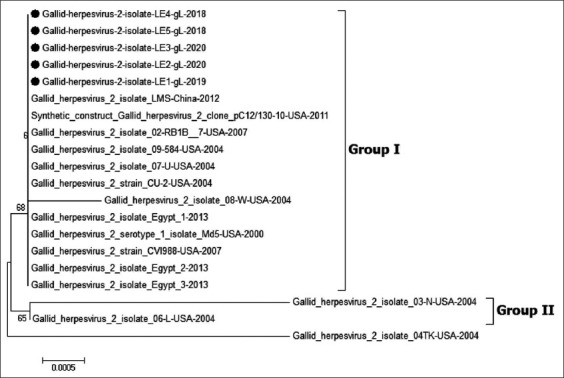
Phylogenetic tree of *gL* gene of Marek disease virus (MDV). The figure shows the phylogenetic analysis of *gL* gene of MDV gene reveling that all Egyptian strains cluster were related to very virulent European strains (ATE, PC12/130). The MDV viruses in our study are indicated with a black dot.

The phylogenetic tree of the *gL* gene also shows it clustered into two major groups (Figure-4). The MDV studied here was related to Group I, consisting of Egyptian and Chinese viruses and vv European viruses. Alternatively, Group 2 comprised vv+ viruses from the USA (TK, X, and N).

### Molecular characterization of the *ICP4* gene in Egyptian viruses

**Figure-5:**
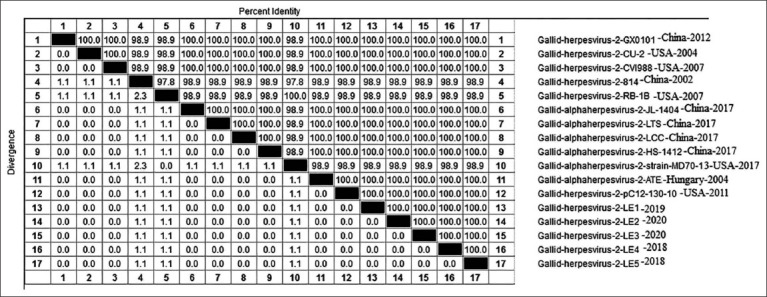
Amino acid (A.A.) identities and divergence of *ICP4* gene of sequenced viruses compared to other selected strains from European, China, and American strains. The figure shows comparative alignment of *ICP4* gene showed that *ICP4* A.A. identity percent of 100% homology, 98.9% to very virulent USA strain RB1B and MD70 and 100% for mild virulent and vaccinal strains.

From the perspective of the *ICP4* phylogeny, the Egyptian viruses studied were genetically related to prototype vv European and Chinese viruses (Y.A., ATE, PC12/130, and GX070060), respectively, with 100% homology, and to the vv USA viruses (RB1B and MD70), with a 98.9% homology and to mildly virulent and vaccinal strains; (CU2 and CV198), with 100% homology (Figure-5). No mutation was detected compared to the Gallid herpesvirus-2 strain ATE from China.

**Figure-6:**
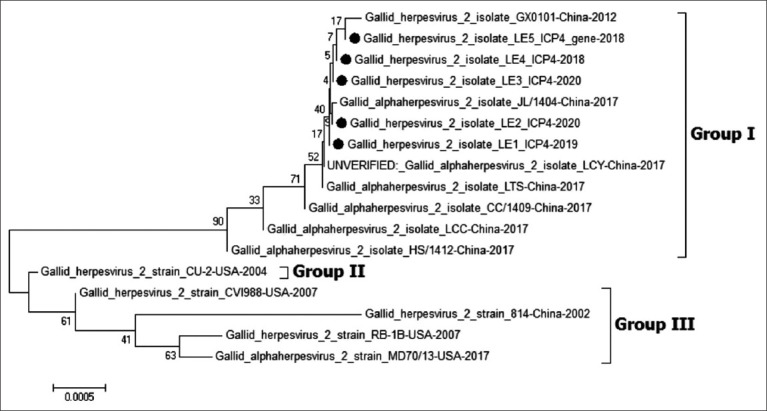
Phylogenetic tree of *ICP4* gene of Marek disease virus (MDV). The figure shows the phylogenetic analysis of *ICP4* gene of MDV gene reveling that all Egyptian strains cluster were related to very virulent European strains (YA, ATE, PC12/130). The MDV viruses in our study are indicated with a black dot.

For phylogenetic analysis, the *ICP4* was classified into three groups. The Egyptian viruses of this study were related to those classified into Group I, including the Chinese viruses (LMS, Y.A., WS03, and GX070060) and the vv European viruses (AT-2539 and PC12/130). Group II comprised the virulent and vv viruses isolated from the USA, whereas Group III contained the mildly virulent viruses; CV1988 and CU-2 (Figure-6).

### GenBank accession numbers

Sequences generated in this study were submitted to GenBank. *Meq*: MT748026- MT748030. *ICP4*: MT748031-MT748035 and *gl*: MT748036-MT748040 (Table-2).

**Table-2:** The accession number of sequenced samples.

No.	Name of sequenced samples	Accession number meq	Accession number ICP4	Accession number GL
1.	Gallid-herpesvirus-2-isolate-LE1-ICP4	MT748026	MT748031	MT748036
2.	Gallid-herpesvirus-2-isolate-LE2-ICP4	MT748027	MT748032	MT748037
8.	Gallid-herpesvirus-2-isolate-LE3-ICP4	MT748028	MT748033	MT748038
9.	Gallid-herpesvirus-2-isolate-LE4-ICP4	MT748029	MT748034	MT748039
14.	Gallid-herpesvirus-2-isolate-LE5-ICP4	MT748030	MT748035	MT748040

The table shows the accession number of *Meq, ICP4, and gL* genes.

## Discussion

Marek’s disease, caused by the MDV, is a highly infectious neoplastic chicken disease that is preventable with vaccination. Although Egyptian hatcheries adopt an intense policy of MDV vaccination using CV1988 or CV1988+HVT at 1-day of age, several cases of vaccine failure have been reported recently [32,38,39]. The appearance of new virulent MDV viruses accounts for the MDV vaccine failure [3]. Transcriptional activity of MDV in tumor cells is also limited to repeat regions, including viral telomerase RNA, viral *IL-8*, *Meq, pp38, and ICP4* [40-42]. The *Meq, IL-8*, and *ICP4* genes are reportedly associated with virulence [42] that evolved more rapidly than other DNA viruses [18]. Nevertheless, literature regarding the molecular composition and evolution of MDV viruses that circulate in Egypt is limited. In this study, we identified circulating MDV in eight governorates from Egypt and studied the molecular evolution of the field strains of MDV by sequencing the *Meq*, *ICP4*, and *gL* genes.

In this study, 40 samples were collected from different provinces in Egypt. Marek’s disease virus appeared in 30 samples by PCR, five of which were sequenced for *Meq, gL, and ICP4* genes. The molecular analysis of *Meq*, *gL*, and *ICP4* genes in 5 MDVs was related to the vv European strains, ATE, and PC12/130, with high homology (99.2-99.4%, 99.7%, and 100%, respectively), as previously described by Mitra *et al*. [29], Hassanin *et al*. [37], Zanaty *et al*. [43]. The *Meq* gene was then used to determine the virulence of MDV strains by the point mutations detected. Studies have shown that these mutations can increase the virus’s increased virulence and oncogenicity [20]. Transactivation of *Meq* can also result in cell transformation [44,45]. Phylogenetic analysis of *Meq* classified MDV into three groups (vv European and Chinese strains, mild virulent, vaccinal strains, and American strains) [37]. The Egyptian strains presented in this study were related to vv European strains, as previously described by Hassanin *et al*. [37]. They acquired new mutations divided into two subgroups A and B. The strains in this study related to subgroup A possessed specific characteristic mutations.

The lengths of the *Meq* gene differ between vaccinal strains (CV1988, 814, and 3004), mild strains (CU-2), and virulent strains. In mild and vaccinal strains, *Meq* was 177 bp longer than in virulent MDV strains (a 59-A.A. insertion), which inhibited the expression of the *Meq* protein [22,46]. All strains in this study had short *Meq* gene sequences, confirming the virulence of field MDVs. Several A.A. point mutations were detected in the *Meq* gene of Egyptian viruses in our study, similar to those identified in a previously isolated Egyptian strain, but with an additional new specific mutation at R176A that correlates with the virulence of MDV (as previously recorded for vv+ MDVs) [13].

The A.A. identity of the five MDVs in this study showed low homology with CVI988 and 3004 (82.5%). This low outcome is due to vaccination failure and outbreaks in Egypt despite intensive vaccination programs with the 3004 and CVI988 strains, as previously described by Lebdah *et al*. [28], Hassanin *et al*. [37], Zanaty *et al*. [43]. Further studies are, therefore, required to evaluate the efficacy of vaccines against vv strains, which breakout Egypt.

MDV *gL* is important for entering viruses into host cells and transmission between cells [26]. Phylogenetic analysis of *gL* in the current study divided strains into two groups (I and II). Group I contained vv European strains, Chinese strains, and mild virulent strains, and Group II comprised American strains [37]. The Egyptian strains were related to Group I, as previously recorded by Hassanin *et al*. [37]. The previous studies also detected a deletion in the *gL* gene cleavage site in vv+ strains isolated from the USA, hypothesized to perform an important role in increased virulence and pathogenicity of the virus [13]. In this study, the deletion was not detected in any virus.

The *ICP4* gene plays an important role in MDV replication [47,48] and virus attenuation by acquiring multiple non-synonymous mutations during *in vitro* serial passages [49]. Phylogenetic analysis of the *ICP4* gene also classified MDV into three groups (vv European and Chinese strains, mild and vaccinal strains, and American strains) as previously recorded [50]. Our MDV in this study clustered to the European/Chinese groups. A previous study reported HNGS101 isolated in China, contained multiple substitutions that altered the function of *ICP4*, decreasing the virulence and pathogenicity of the virus [51]. This study detected no virus mutations in *ICP*4 gene.

## Conclusion

Twenty samples in our study were positive for MDV. The Egyptian strains in this study were related to the very virulent European viruses (Gallid herpesvirus 2 ATE and PC12/30). They were clustered into new subgroups A and B by phylogenetic analysis distinct from the vaccine strains. Further studies should evaluate the protection contributed by current vaccines used in Egypt.

## Authors’ Contributions

HSE and SEO collected samples. NY, AE, and FA: Detection and molecular characterization of samples. All authors were involved in the writing, analysis of the data, and reviewed the manuscript. All authors read and approved the final manuscript.
